# Return to work after sepsis—a German population-based health claims study

**DOI:** 10.3389/fmed.2023.1187809

**Published:** 2023-05-25

**Authors:** Carolin Fleischmann-Struzek, Bianka Ditscheid, Norman Rose, Melissa Spoden, Lisa Wedekind, Peter Schlattmann, Christian Günster, Konrad Reinhart, Christiane S. Hartog, Antje Freytag

**Affiliations:** ^1^Institute of Infectious Diseases and Infection Control, Jena University Hospital, Jena, Germany; ^2^Center for Sepsis Control and Care, Jena University Hospital/Friedrich Schiller University Jena, Jena, Germany; ^3^Institute of General Practice and Family Medicine, Jena University Hospital, Jena, Germany; ^4^Research Institute of the Local Health Care Funds, Berlin, Germany; ^5^Federal Association of the Local Health Care Funds, Berlin, Germany; ^6^Institute of Medical Statistics, Computer and Data Sciences, Jena University Hospital, Jena, Germany; ^7^Department of Anesthesiology and Operative Intensive Care Medicine, Charité Universitätsmedizin Berlin, Berlin, Germany; ^8^Klinik Bavaria, Kreischa, Germany

**Keywords:** sepsis, return to work, survivor, septic shock, post-sepsis-syndrome

## Abstract

**Background:**

Long-term impairments after sepsis can impede the return to work in survivors. We aimed to describe rates of return to work 6 and 12 months postsepsis.

**Methods:**

This retrospective, population-based cohort study was based on health claims data of the German AOK health insurance of 23.0 million beneficiaries. We included 12-months survivors after hospital-treated sepsis in 2013/2014, who were ≤60 years at the time of the admission and were working in the year presepsis. We assessed the prevalence of return to work (RTW), persistent inability to work and early retirement.

**Results:**

Among 7,370 working age sepsis survivors, 69.2% returned to work at 6 months postsepsis, while 22.8% were on sick leave and 8.0% retired early. At 12 months postsepsis, the RTW rate increased to 76.9%, whereas 9.8% were still on sick leave and 13.3% retired early. Survivors who returned to work had a mean of 70 (SD 93) sick leave days in the 12 months presepsis (median 28 days, IQR 108 days).

**Conclusion:**

One out of four working age sepsis survivors does not resume work in the year postsepsis. Specific rehabilitation and targeted aftercare may be opportunities to reduce barriers to RTW after sepsis.

## Introduction

An estimated 38 million patients survive sepsis every year (1). While sepsis is often considered as a disease of the elderly, research suggests that more than one third of sepsis survivors is aged <65 years in high-income countries (2). Sepsis can lead to long-term sequelae with devastating consequences in survivors, hampering the return to normal living even months and years after the acute disease (3, 4). Although cognitive impairments are less incident among younger survivors ≤65 years, they suffer particularly more often from new mental health impairments compared to older sepsis survivors (5). In addition, more than half of younger sepsis survivors were found to develop new medical diagnoses in the year postsepsis (5). These sequelae pose barriers to successful recovery (4), including the ability to return to work (6). In a Danish cohort study among ICU-treated septic shock survivors, only 43% of previously working patients had resumed employment one year after hospital discharge (6). Such delays in return to work can negatively impact physical and mental health (7), and have relevant financial implications for patients, families and the society (8).

Therefore, return to work can be considered as a major patient-relevant outcome after sepsis and serve as indicator of recovery. To date, however, data on the adverse change in employment status after sepsis is scarce, particularly in cohorts of non-ICU-treated sepsis patients, as most existing studies on return to work focus on cohorts of general ICU survivors (9). We therefore aimed to (1) assess the prevalence of return to work 6 and 12 months after postsepsis; (2) quantify the duration of sick leave, and (3) compare the health status of patients with vs. without return to work.

## Materials and methods

The Institutional Review Board of the Friedrich Schiller University Jena approved this study (2019-1282-Daten, date: 2019-01-17, study title: “sepsis: long-term sequelae, risk factors, health care utilization and costs”). The requirement for informed consent was waived because all data were deidentified. This study was reported according to the Strengthening the Reporting of Observational Studies in Epidemiology (STROBE) reporting guideline and followed the Helsinki Declaration of 1975.

### Database

We conducted a population-based cohort study using nationwide health claims data of the German AOK health insurance. The AOK health insurance is the largest health insurance in Germany and covers around 30% of the German population (10). AOK health claims data contain de-identified information on patient demographics and working status, hospitalizations, outpatient visits, outpatient drug prescriptions, rehabilitation, nursing care dependency, and sick leave days.

### Patient sample

Among insurance beneficiaries, we identified working adults ≤60 years who were treated in hospital with sepsis between January 2013 and December 2014. Sepsis was identified using explicit ICD-10-codes for sepsis (see Supplementary material) coded as primary or secondary hospital discharge diagnoses, including severe sepsis and septic shock according to the sepsis-1/2 definition (11, 12). Working status is recorded on a quarterly basis in health insurance data. We defined working adults by the following categories: mandatory/voluntary AOK-insured employees, AOK-insured employees in rehabilitation or AOK-insured employees applying for pension payments in the four quarters prior to the hospital admission with sepsis. We excluded beneficiaries who were hospitalized with sepsis in the 24 months prior to hospital admission, and beneficiaries not consecutively enrolled in the insurance for 12 months prior and 36 months after sepsis hospitalization or until death. In the observation period, the first sepsis hospitalization was denoted as index hospitalization.

### Outcomes

We investigated the following outcomes 6 and 12 months after discharge from the index hospitalization among 12-months survivors of sepsis: return to work (<180/360 days of sick leave among mandatory/voluntary AOK-insured employees and AOK-insured employees in rehabilitation), persistent inability to work (≥180/360 days of sick leave among mandatory/voluntary AOK-insured employees and AOK-insured employees in rehabilitation) and early retirement (working status: applied for pension payment or received pension payment). Among patients who returned to work, we assessed the length of sick leave after sepsis hospitalization. Furthermore, we compared patients with return to work vs. patients without return to work (inability to work or early retirement) regarding demographics, characteristics of the acute sepsis disease and treatment and postsepsis morbidity. We considered new cognitive, medical and psychological diagnoses in the 12 months after discharge as postsepsis morbidity as previously described in the SEPFROK study and quantified their co-occurrence (5).

### Statistical analyses

We report proportions with 95% confidence intervals (CI), means with standard deviation (SD) and medians with interquartile ranges (IQR). We analyzed all outcomes for the total population of hospital survivors and the subgroups of severe sepsis and non-severe sepsis survivors (identified by presence/absence of ICD-10-GM codes R65.1 and R57.2, respectively), ICU-treated and non-ICU-treated sepsis survivors (identified by presence/absence of operation and procedural codes for intensive care complex treatment, see Supplementary material), survivors with and without presepsis medical, psychological and cognitive impairments [see SEPFROK study (5)] and by age groups (<40, 40–49, 50–60). Patients with and without return to work were compared by chi-square tests (dichotomous variables) and Welch tests (metric variables). We conducted all analyses using SAS Version 9.4 and R Version 4.1.2 (13).

## Results

### Patient characteristics

**Figure 1:**
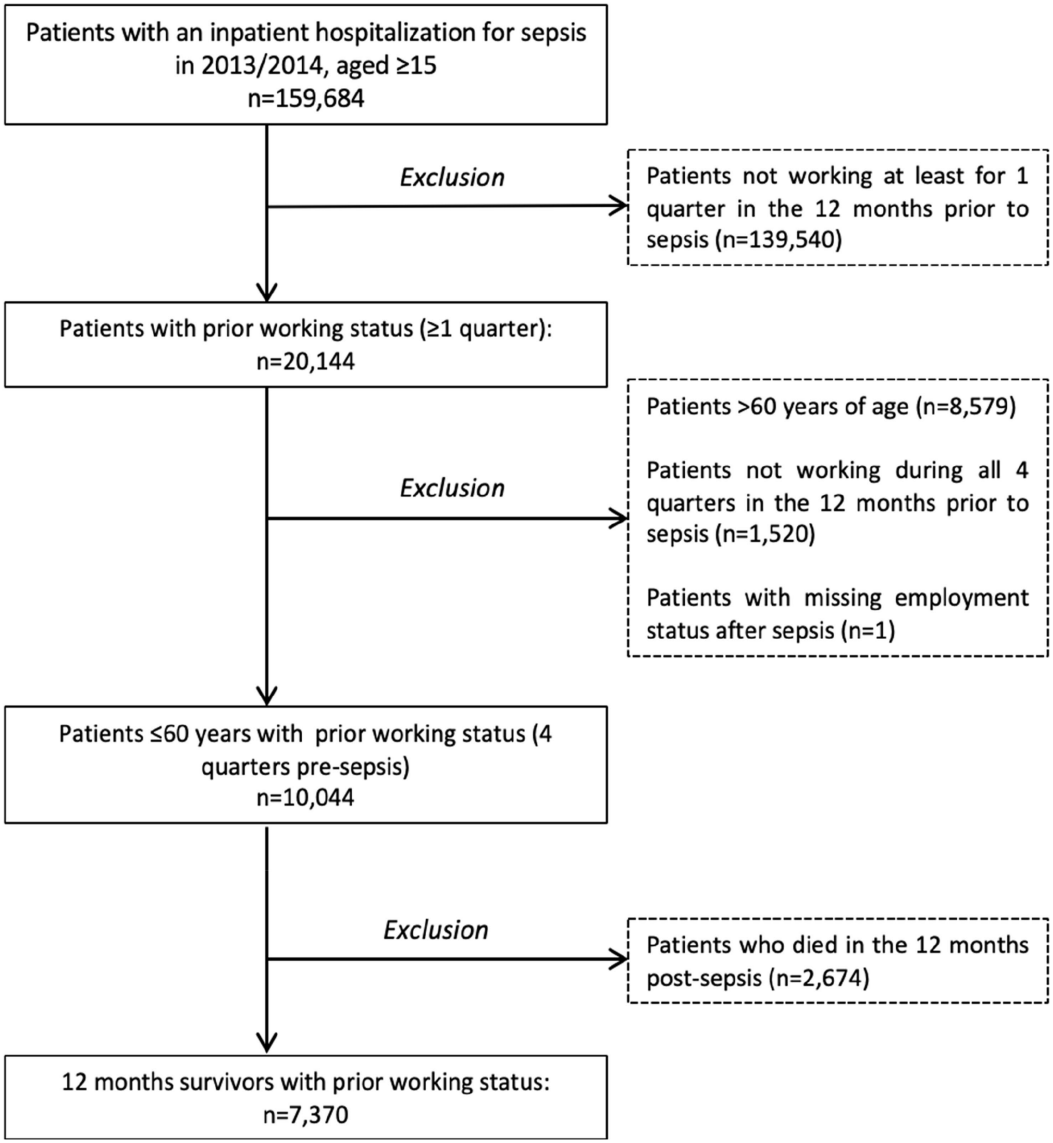
Flow of study inclusion.

Among 23 million AOK beneficiaries in 2013/2014, we identified 10,044 working adults with sepsis hospitalization. 7,370 (73.4%) survived 12 months after discharge (Figure 1). Mean age of survivors was 49 years (SD 10) and 35.3% were female (Supplementary Table S1). 35.7% of survivors had no prior comorbidity, while 26.5% had one, 33.5% had two to four and 4.3% had more than four prior comorbidities in the year presepsis. Among survivors with comorbidities, chronic pulmonary diseases, diabetes and cancer were most common and affected 24.3, 18.5 and 16.5% of survivors, respectively (Supplementary Table S1). 2,257 (30.6%) of patients had severe sepsis and 2,411 (32.7%) were treated in ICU. Pulmonary infections were the most common focus of infection (32.6% of patients) followed by genitourinary infections (24.3%) and abdominal infections (15.5%). 55.4% of patients were admitted as emergency and 39.1% received surgical treatment. Mean hospital length of stay was 22 days (SD 24). Most patients were discharged home (80.1%), while 11.1% were discharged to other hospitals, 5.1% to rehabilitation and 1.6% to a skilled nursing facility (Supplementary Table S1).

### Return to work (RTW) after sepsis

**Figure 2:**
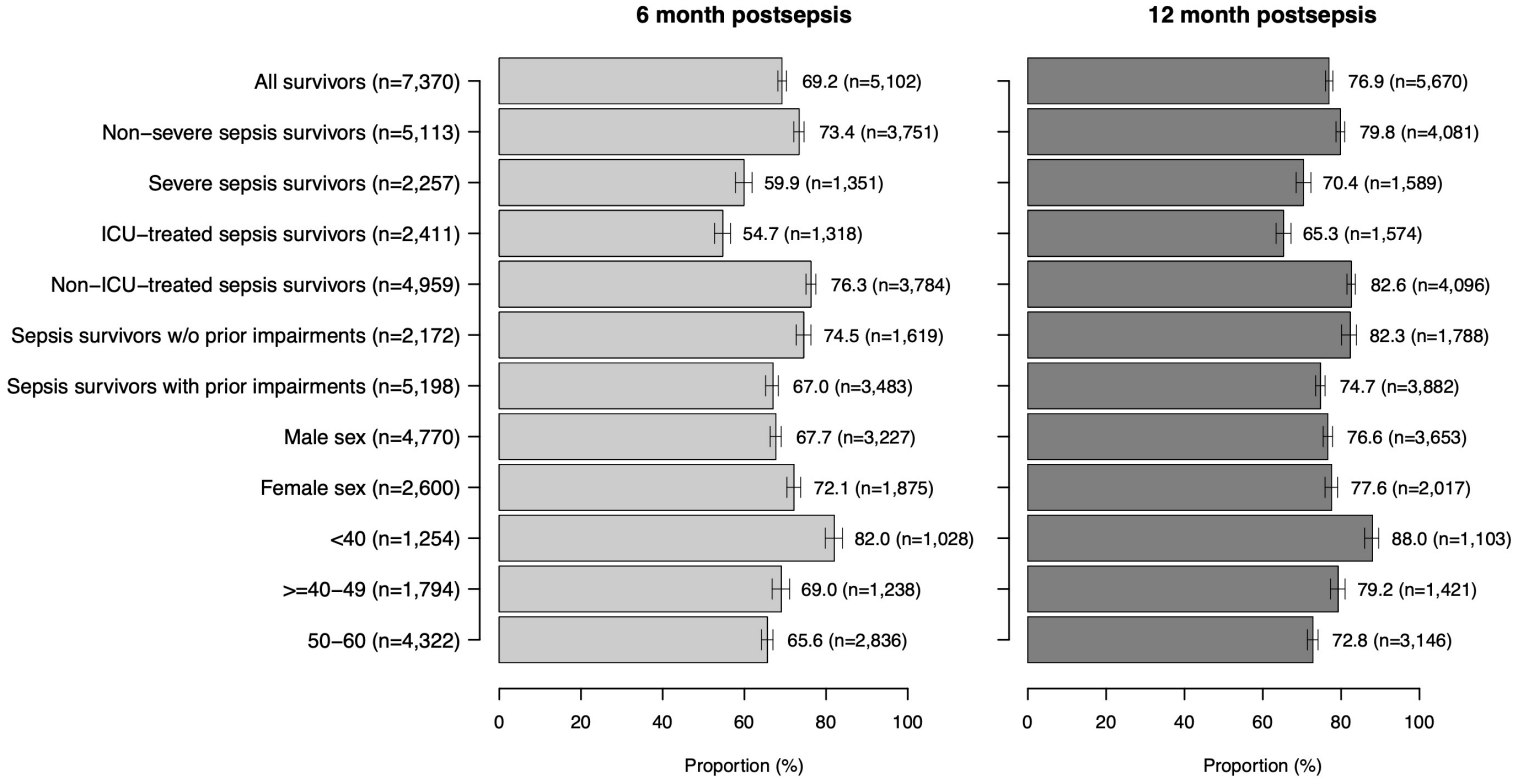
Return to work rates among all and subgroups of patients at 6 and 12 months post-sepsis.

**Figure 3:**
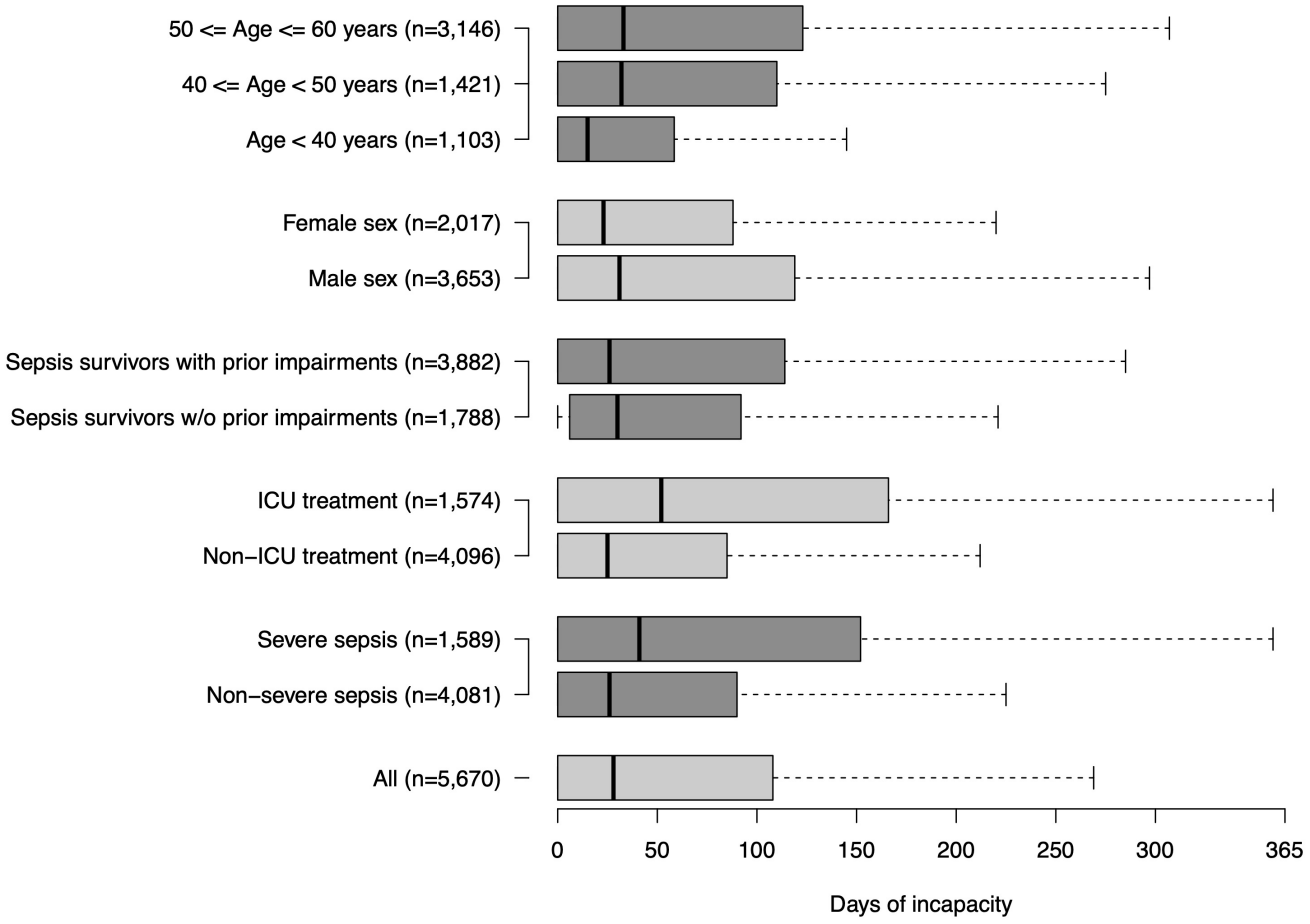
Duration of sick leave among all sepsis patients and subgroups in the first year after sepsis.

RTW rates 6 and 12 months after sepsis were 69.2 and 76.9%, respectively (Figure 2). The proportion of survivors on sick leave declined from 22.8% at 6 months to 9.8% at 12 months (Supplementary Table S2). The proportion of survivors, who retired early, rose from 8.0 to 13.3% in this time frame. Survivors who returned to work had a mean of 70 sick leave days (SD 93) in the 12 months postsepsis (median 28 days, IQR 108 days, Figure 3).

Among subgroups (ICU-/non-ICU sepsis patients, severe/non-severe sepsis patients, patients with/without prior impairments, age groups), survivors with ICU-treated sepsis had the lowest 12-months RTW rates (65.3%), while the highest proportion of survivors was on sick leave or retired early in this subgroup (Figure 2). They also had the highest number of sick leave days among RTW survivors (mean 91 (105) days, median 52 (IQR 166) days, Figure 3). Younger sepsis survivors <40 years most frequently returned to work among all subgroups analyzed (12-months RTW rate: 88.0%), however, also 7.6% of these patients remained persistently unable to work and 4.5% retired early (Supplementary Table S2).

### Comparison of survivors with vs. without return to work

**Figure 4:**
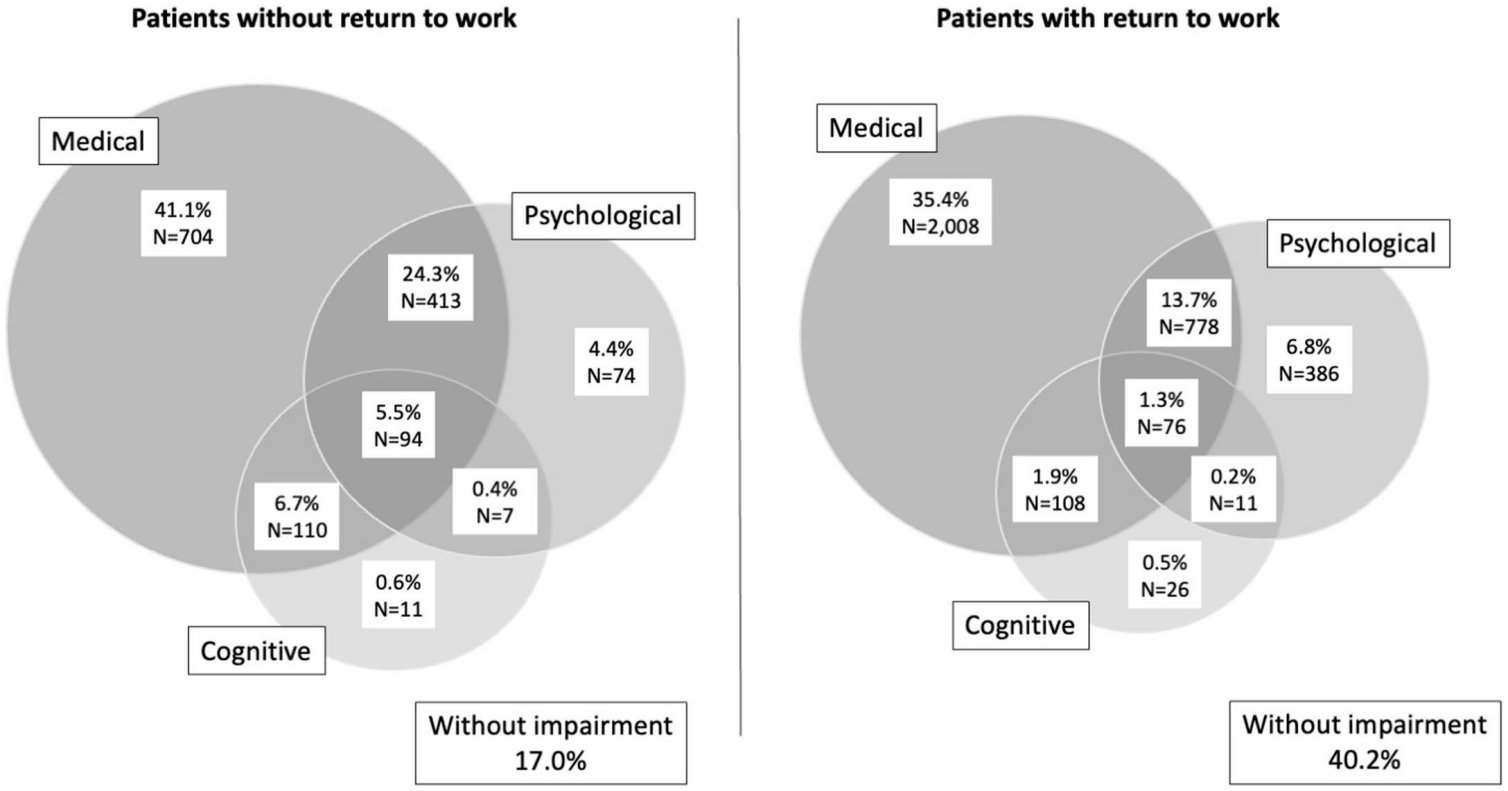
Overlap in cognitive, medical and psychological impairments in survivors without **(left)** and with **(right)** return to work 12 months after sepsis.

We compared 5,670 survivors with RTW 12 months after sepsis with 1,700 survivors without RTW. Patients with RTW were younger (mean age 48 years (SD 10) vs. 51 years (SD 8), *p* < 0.001) and had fewer pre-existing comorbidities (1.3 (SD 1.5) vs. 1.8 (1.6), *p* < 0.001, Table 1). The gender distribution was not significantly different between groups. Patients who returned to work 12 months post-discharge had less often severe sepsis (28.0% vs. 39.3%, *p* < 0.001) and were less frequently treated in ICU (27.8% vs. 49.2%, *p* < 0.001). Furthermore, their infection onset was less often hospital-acquired (15.1% vs. 32.6%, *p* < 0.001). In the 12 months postsepsis, survivors with RTW had a lower burden of postsepsis morbidity. A lower proportion of RTW survivors had at least one new medical, cognitive or psychological diagnosis (59.8% vs. 83.1%, *p* < 0.001) and medical, cognitive and psychological diagnoses were less frequently overlapping (Figure 4). Comparing survivors with and without RTW, 4.1% vs. 13.8% had a new cognitive diagnosis, 52.4% vs. 77.7% had a new medical diagnosis and 22.1% vs. 34.6% had a new psychological diagnosis (all *p* < 0.001). Survivors with RTW had less new nursing care degrees compared to survivors without RTW (4.8% vs. 25.1%, p < 0.001). Furthermore, they were less frequently readmitted to hospital (1.2 (SD 1.9) vs. 2.7 (SD 3.0) readmissions/year, *p* < 0.001) and had a lower mean number of outpatient contacts (35.2 (SD 40.0) vs. 56.8 (SD 49.6) contacts/year, *p* < 0.001).

**Table 1:** Comparison of characteristics of patients with vs. without RTW 12 months after sepsis.

Characteristics	Patients with RTW	Patients without RTW	*p*
Number of 12-months survivors	5,670	1,700	
Age at index admission, mean (SD)	48 (10.2)	51.3 (8.1)	<0.0001
Female sex, %. (95% CI)	35.6 (34.3–36.8)	34.3 (32.1–36.6)	0.348
Presepsis CCI, mean (SD)	1.3 (1.5)	1.8 (1.6)	<0.0001
Index hospitalization			
Severe sepsis, % (95% CI)	28.0 (26.9–29.2)	39.3 (37.0–41.6)	<0.0001
Septic shock, % (95% CI)	6.8 (6.1–7.5)	12.0 (10.5–13.6)	<0.0001
ICU-treatment, % (95% CI)	27.8 (26.6–28.9)	49.2 (46.9–51.6)	<0.0001
Hospital-acquired infection, % (95% CI)	15.1 (14.2–16.1)	32.6 (30.5–34.9)	<0.0001
Multi-resistant infection, % (95% CI)	2.6 (2.3–3.1)	5.6 (4.6–6.8)	<0.0001
Discharge to rehabilitation, % (95% CI)	2.8 (2.4–3.3)	12.8 (11.3–14.4)	<0.0001
Hospital length of stay in days, mean (SD)	18.2 (19.7)	34.5 (33.2)	<0.0001
Sepsis sequealae			
New cognitive impairment, % of at risk (95% CI)	4.1 (3.6–4.7)	13.8 (12.2–15.5)	<0.0001
New psychological impairment, % (95% CI)	22.1 (21.0–23.2)	34.6 (32.4–36.9)	<0.0001
New medical impairment, % (95% CI)	52.4 (51.1–53.7)	77.7 (75.7–79.6)	<0.0001
New nursing care grade, % of at risk (95% CI)	4.8 (4.2–5.4)	25.1 (23.0–27.3)	<0.0001
New nursing home resident, % of at risk (95% CI)	1.8 (1.4–2.1)	7.2 (6.1–8.5)	<0.0001
Treatments postsepsis			
Number of readmissions 30d after discharge, mean (SD)	0.19 (0.39)	0.36 (0.48)	<0.0001
Number of readmissions within 12 months after discharge (inpatient), mean (SD)	1.18 (1.92)	2.73 (2.99)	<0.0001
Number of outpatient consultations (contacts), mean (SD)	35.2 (40.0)	56.8 (49.6)	<0.0001

## Discussion

In this population-based cohort study among 7,370 working age sepsis survivors, we found three out of four previously working sepsis survivors returned to work in the 12 months after sepsis, while a considerable proportion of survivors was still on sick leave (9.8%) or retired prematurely (13.3%). Return to work rates decreased with age, but even among younger survivors and patients with sepsis without organ dysfunction or non-ICU-treated sepsis, a considerable proportion of survivors did not resume work after 1 year. In the age group <40 years, more than 10% of survivors were on sick leave or had retired early 1 year after the acute disease. Such adverse changes in working status can have substantial impact on patients and relatives, including impaired subjective well-being and life satisfaction (14, 15), the risk of new mental health impairments (7), and substantial loss earnings (16). Among survivors who returned to work in the 12 months postsepsis, 59.8% had new cognitive, psychological or physical diagnoses, which suggests that also these survivors may be impaired in their work force, cannot pursue the same work as presepsis and may have special requirements on workplace environments. This may negatively impact their quality of life, for which reintegration into normal living, including return to work, was identified a key domain (3). Furthermore, it may increase the financial burden of families and caregivers (17) and the direct and indirect socioeconomic impact of sepsis (18).

Previous estimates on return to work after sepsis and critical illness were mainly derived from smaller surveys among ICU survivors, which found lower rates of return to work in these more severely affected patients according to a recent meta-analysis (pooled RTW rate: 60% of 12-months survivors after critical illness), although RTW rates varied widely between studies (16). However, lower return to work rates were found ICU survivors with multiple organ dysfunction (19). In line with this, only 43% of septic shock survivors reported that they have returned to work 12 months postsepsis in a cohort of ICU-treated septic shock survivors in Denmark (6), while 77% of survivors reported no change in employment status 3.5 years postsepsis in a Canadian cohort study (20). Notably, return to work rates did not differ significantly between sepsis and non-sepsis ICU survivors according to the results of a Australian prospective multicenter cohort study (44.1 vs. 40.4% with unemployment due to health at 6 months post-discharge, respectively) (21). Ours are also on the upper limit of observed return to work rates among Covid-19 survivors, which in approximately one third of cases were found to be affected by sepsis (77.9% of ICU-treated Covid-19 patients) (22). Among ICU-treated Covid-19 survivors, between 11.4% (23) and 43.3% (24) were unable to resume work 6 months postsepsis according to a recent systematic review (45.3% in our study) (25). Lower rates were found among mixed cohorts of ICU- and non-ICU-treated Covid-19 survivors, with, e.g., 8.2% of patients without return to work among hospital-treated Covid-19 survivors in Switzerland (26). Differences in health care systems, e.g., the existence of sick leave compensations (27), and ICU-admission policies and capacities, as well as in disease severity, for example the proportion of patients receiving mechanical ventilation, may contribute to the differences in return to work in the observed studies. Furthermore, differences in place of living of patients may influence also return to work outcomes, as a broader access to rehabilitation was found to exist in predominantly urban locations (28). Notably, our study has a population-based design including an unselected cohort of ICU and non-ICU treated sepsis survivors of all severities and was based on the record of working status in health claims data, which may contribute to the relatively lower return to work rates estimates in comparison to other studies, especially among ICU-treated sepsis survivors.

Although we are unable to determine the underlying reasons for the observed changes in working status, we found that survivors with adverse change in working status had a higher comorbidity burden prior to sepsis, and also more frequently suffered from new cognitive, psychological and medical diagnoses after the acute septic disease, which is consistent with previous research (16, 29, 30). Particularly new cognitive impairments and fatigue were identified as major barriers to return to work, but also persistent frailty and a loss of confidence in the own competencies and abilities (30). This may open opportunities for targeted interventions to facilitate the return to work by addressing postsepsis impairments through interventions specifically targeted towards the need of working age sepsis survivors, such as specialized inpatient and outpatient rehabilitation. In our cohort, only 5.1% of working-age sepsis survivors were discharged to rehabilitation facilities, which is a very low proportion compared to other acute diseases, e.g., stroke, after which 54.4% of patients and 85.1% of all patients in a primary target group for rehabilitation underwent rehabilitation in Germany (31). Furthermore, programs for work reintegration, workplace adjustments, and improved awareness towards the needs and long-term impairments of survivors among care providers and employers were factors that facilitated the return to work after stroke (32, 33) and may serve as examples to support postsepsis return to work. Particularly, this could include reintegration programs for older employees, as they are predominantly affected by postsepsis impairments (5).

Our study has several potential limitations. First, we were unable to differentiate involuntary from voluntary change in working status and to judge patient satisfaction with potential changes, which is important to consider in the interpretation of our data. In a Canadian study among sepsis survivors, 80% of sepsis survivors were very or mostly happy with their quality of life, although 75% reported working less or not, all compared to presepsis (20). Second, we did not capture any changes in working time hours as one aspect of adverse change in working status. Third, the notification of a change in working status to the health insurance may be delayed in some cases, which may explain the fact that among survivors with RTW according to health claims data, 4.8% were dependent on nursing care. Fourth, the identification of sepsis patients in health claims data suffers from limited sensitivity and may miss a certain proportion of sepsis cases (34, 35), thus may confound also the estimates of RTW rates among survivors. This also applies to postsepsis diagnosis, for which the validity of diagnosis in health claims data remains mostly unknown. Fifth, our results did not emphasize the current sepsis-3 definition (36), as in 2013/2014, sepsis was defined according to the sepsis-1/2 definition (11, 12) in Germany. Severe sepsis cases denote patients with sepsis-related organ dysfunction. Sixth, our observation period was limited to 12 months in this study, however, RTW may also occur after this period (16).

## Conclusion

Sepsis impedes the return to work in working-age sepsis survivors. Specific rehabilitation and targeted aftercare may be opportunities to reduce barriers to RTW after sepsis. Given the tremendous implications that the change in working status may have, return to work must be considered as important patient-relevant outcome in future studies on effective treatments during and after sepsis.

## Data availability statement

The data analyzed in this study is subject to the following licenses/restrictions: The authors confirm that the data utilized in this study cannot be made available in the manuscript, the Supplementary material, or in a public repository due to German data protection laws (“Bundesdatenschutzgesetz,” BDSG). Therefore, they are stored on a secure drive in the AOK Research Institute (WIdO), to facilitate replication of the results. Generally, access to data of statutory health insurance funds for research purposes is possible only under the conditions defined in German Social Law (SGB V § 287). Requests for data access can be sent as a formal proposal specifying the recipient and purpose of the data transfer to the appropriate data protection agency. Access to the data used in this study can only be provided to external parties under the conditions of the cooperation contract of this research project and after written approval by the sickness fund. For assistance in obtaining access to the data, please contact wido@wido.bv.aok.de.

## Ethics statement

The studies involving human participants were reviewed and approved by The Institutional Review Board of the Friedrich Schiller University Jena (2019-1282-Daten, date: 2019-01-17). Written informed consent for participation was not required for this study in accordance with the national legislation and the institutional requirements.

## Author contributions

CF-S, BD, and AF conceptualized the study and drafted the data analysis plan. CH, CG, KR, LW, and PS too part in the design of the underlying SEPFROK study. MS and BD prepared and checked the data. BD and NR conducted the statistical analyses. CF-S wrote the first draft of the manuscript. AF and BD completed the first draft of the manuscript. All authors contributed to the article and approved the submitted version.

## Funding

The study was funded by the German Innovations Fund of the Federal Joint Committee in Germany (G-BA) (grant number: 01VSF17010).

## Publisher’s note

All claims expressed in this article are solely those of the authors and do not necessarily represent those of their affiliated organizations, or those of the publisher, the editors and the reviewers. Any product that may be evaluated in this article, or claim that may be made by its manufacturer, is not guaranteed or endorsed by the publisher.
