# Supplementary material for: Return to work after sepsis—a German population-based health claims study
Source: Front Med (Lausanne). 2023 May 25;10:1187809. doi: 10.3389/fmed.2023.1187809 (PMC10248449; doi:10.3389/fmed.2023.1187809)
Supplement: Supplementary file 1 [file Data_Sheet_1.docx]

**Supplement to**

**Return to work after sepsis – A German population-based health claims study**

Fleischmann-Struzek Carolin,^1,2^ Ditscheid Bianka,^3^ Rose Norman, ^1,2^ Spoden Melissa,^4^ Wedekind Lisa,^5^ Schlattmann Peter,^5^ Günster Christian,^4^ Reinhart Konrad,^6^ Hartog Christiane S,^6,7^ Freytag Antje.^3^

**Content**

ICD-Codes and Definitions – page 3

Supplement Table S1 – page 21

**ICD-Codes and Definitions**

**Sepsis case identification**

The identification of sepsis patients in our study was based on the following ICD-10-GM codes in health claims data. At least one of the codes had to be coded as primary and secondary discharge diagnosis. During the complete observation period, sepsis coding was based on the sepsis-1/2 criteria in Germany. Therefore, severity was classified as: sepsis – all forms; severe sepsis (including septic shock); septic shock; and non-severe sepsis.

| Sepsis |  |
| --- | --- |
| ICD-10-GM Codes: |  |
| A02.1 | Salmonella sepsis |
| A20.0 | Bubonic plague |
| A20.7 | Septicaemic plague |
| A21.7 | Generalized tularaemia |
| A22.7 | Anthrax sepsis |
| A24.1 | Acute or fulminating melioidosis |
| A26.7 | Erysipelothrix sepsis |
| A28.2 | Extraintestinal yersiniosis |
| A32.7 | Listerial sepsis |
| A39.1 | Waterhouse-Friderichsen syndrome |
| A39.2 | Acute meningococcal sepsis |
| A39.3 | Chronic meningogoccal sepsis |
| A39.4 | Meningococcaemia, unspecified |
| A40 | Streptococcal sepsis |
| A41 | Other sepsis |
| A42.7 | Actinomycotic sepsis |
| A48.3 | Toxic shock syndrome |
| A49.9 | Bacterial infection, unspecified |
| A54.8 | Other gonococcal infections |
| B00.7 | Disseminated herpesviral disease |
| B37.6 | Candidal endocarditis |
| B37.7 | Candidal sepsis |
| B49 | Unspecified mycosis |
| O75.3 | Other infection during labour |
| O85 | other puerperal infections |
| R65.0 | Systemic Inflammatory Response Syndrome of infectious origin without organ failure |
| R65.1 | Systemic Inflammatory Response Syndrome of infectious origin with organ failure |
| R57.2 | Septic shock |

**Patient description**

Comorbidities were defined in accordance with Quan et al.^25^ based on inpatient and outpatient diagnosis in the year pre-sepsis. An unweighted Charlson Comorbidity Index was calculated.

The following data were used for description of the index hospitalization and the definition of subgroups:

| Emergency admission at index hospitalization | Admission type: referral by physician or other (1,2,5), emergency (4), transfer from other hospital (3). |
| --- | --- |
| Level of care in the year prior to index hospitalization | A care level/grade describes a person's individually recognized need for care and assistance in accordance with the German Care Insurance Act (SGB XI) and thus determines the scope of support benefits that could be applied for from the relevant care insurance fund. |

| **Focus of infection** |  |
| --- | --- |
| Respiratory tract |  |
| ICD-10-GM Codes: |  |
| J01 | Acute sinusitis |
| J02 | Acute pharyngitis |
| J03 | Acute tonsillitis |
| J04 | Acute laryngitis and tracheitis |
| J06 | Acute upper respiratory infections of multiple and unspecified sites |
| J05 | Acute obstructive laryngitis [croup] and epiglottitis |
| J09 | Influenza due to identified zoonotic or pandemic influenza virus |
| J10 | Influenza due to identified seasonal influenza virus |
| J11 | Influenza, virus not identified |
| J12 | Viral pneumonia, not elsewhere classified |
| J13 | Pneumonia due to Streptococcus pneumoniae |
| J14 | Pneumonia due to Haemophilus influenzae |
| J15 | Bacterial pneumonia, not elsewhere classified |
| J16 | Pneumonia due to other infectious organisms, not elsewhere classified |
| J17 | Pneumonia in diseases classified elsewhere |
| J18 | Pneumonia, organism unspecified |
| J20 | Acute bronchitis |
| J21 | Acute bronchiolitis |
| J22 | Unspecified acute lower respiratory infection |
| J44.0 | Chronic obstructive pulmonary disease with acute lower respiratory infection |
| J44.1 | Chronic obstructive pulmonary disease with acute exacerbation, unspecified |
| J86 | Pyothorax |
| J85 | Abscess of lung and mediastinum |
| A15 | Respiratory tuberculosis, bacteriologically or histologically confirmed |
| A16 | Respiratory tuberculosis, not confirmed bacteriologically or histologically |
| U69.00 | Hospital-acquired pneumonia in other diseases classified elsewhere |
| A36 | Diphtheria |
| A37 | Whooping cough |
| B38 | Coccidioidomycosis |
| B39 | Histoplasmosis |

| Abdominal infections |  |
| --- | --- |
| ICD-10-GM Codes: |  |
| A00 | Cholera |
| A01 | Typhoid and paratyphoid fevers |
| A02 | Other salmonella infections |
| A03 | Shigellosis |
| A04 | Other bacterial intestinal infections |
| A05 | Other bacterial foodborne intoxications, not elsewhere classified |
| A06 | Amoebiasis |
| A07 | Other protozoal intestinal diseases |
| A08 | Viral and other specified intestinal infections |
| A09 | Other gastroenteritis and colitis of infectious and unspecified origin |
| K35 | Acute appendicitis |
| K37 | Unspecified appendicitis |
| K36 | Other appendicitis |
| K57.02 | Diverticular disease of small intestine with perforation and abscess without bleeding |
| K57.03 | Diverticular disease of small intestine with perforation and abscess with bleeding |
| K57.12 | Diverticular disease of small intestine without perforation or abscess without bleeding |
| K57.13 | Diverticular disease of small intestine without perforation or abscess wit bleeding |
| K57.22 | Diverticular disease of large intestine with perforation and abscess without bleeding |
| K57.23 | Diverticular disease of large intestine with perforation, abscess and bleeding |
| K57.32 | Diverticular disease of large intestine without perforation or abscess wihout bleeding |
| K57.33 | Diverticular disease of large intestine without perforation or abscess wit bleeding |
| K57.42 | Diverticular disease of both small and large intestine with perforation and abscess without bleeding |
| K57.43 | Diverticular disease of both small and large intestine with perforation, abscess and bleeding |
| K57.52 | Diverticular disease of both small and large intestine without perforation or abscess or bleeding |
| K57.53 | Diverticular disease of both small and large intestine without perforation or abscess with bleeding |
| K57.82 | Diverticular disease of intestine, part unspecified, with perforation and abscess without bleeding |
| K57.83 | Diverticular disease of intestine, part unspecified with perforation, abscess and bleeding |
| K57.92 | Diverticular disease of intestine, part unspecified, without perforation, abscess or bleeding |
| K57.93 | Diverticular disease of intestine, part unspecified, without perforation or abscess with bleeding |
| K61 | Abscess of anal and rectal regions |
| K65 | Peritonitis |
| K67 | Disorders of peritoneum in infectious diseases classified elsewhere |
| K63.0 | Abscess of intestine |
| K63.1 | Perforation of intestine (nontraumatic) |
| K75.0 | Abscess of liver |
| K75.1 | Phlebitis of portal vein |
| K81.0 | Cholecystitis |
| K77.0 | Liver disorders in infectious and parasitic diseases classified elsewhere |
| U69.40! | Recurrent infection due to Clostridium difficile |

| Wound/soft tissue infection |  |
| --- | --- |
| ICD-10-GM Codes: |  |
| A46 | Erysipelas |
| B47 | Mycetoma |
| L03 | Phlegmon |
| L04 | Acute lymphadenitis |
| L08 | Other local infections of skin and subcutaneous tissue |
| L05 | Pilonidal cyst |
| B00 | Herpesviral [herpes simplex] infections |
| B07 | Viral warts |
| B08 | Other viral infections characterized by skin and mucous membrane lesions, not elsewhere classified |
| B09 | Unspecified viral infection characterized by skin and mucous membrane lesions |
| H05.0 | Acute inflammation of orbit |
| H60.2 | Malignant otitis externa |
| H70.0 | Acute mastoiditis |
| J36 | Peritonsillar abscess |
| J39.0 | Retropharyngeal and parapharyngeal abscess |
| J39.1 | Other abscess of pharynx |
| L02 | Cutaneous abscess, furuncle and carbuncle |

| Genitourinary system infection |  |
| --- | --- |
| ICD-10-GM Codes: |  |
| N10 | Acute tubulo-interstitial nephritis |
| N15.1 | Renal and perinephric abscess |
| N15.9 | Renal tubulo-interstitial disease, unspecified |
| N34 | Urethritis and urethral syndrome |
| N30 | Cystitis |
| N39.0 | Urinary tract infection, site not specified |
| N41 | Inflammatory diseases of prostate |
| N45 | Orchitis and epididymitis |
| N48.2 | Other inflammatory disorders of penis |
| N49 | Inflammatory disorders of male genital organs, not elsewhere classified |
| N70 | Salpingitis and oophoritis |
| N71 | Inflammatory disease of uterus, except cervix |
| N72 | Inflammatory disease of cervix uteri |
| N73 | Other female pelvic inflammatory diseases |
| N74 | Female pelvic inflammatory disorders in diseases classified elsewhere |
| N75 | Diseases of Bartholin gland |
| N76 | Other inflammation of vagina and vulva |
| N77 | Vulvovaginal ulceration and inflammation in diseases classified elsewhere |
| N61 | Inflammatory disorders of breast |
| N98.0 | Infection associated with artificial insemination |
| A59 | Trichomoniasis |
| A55 | Chlamydial lymphogranuloma (venereum) |
| A56 | Other sexually transmitted chlamydial diseases |

| Central nervous system infection |  |
| --- | --- |
| ICD-10-GM Codes: |  |
| A39 | Meningococcal infection |
| G00 | Bacterial meningitis, not elsewhere classified |
| G01 | Meningitis in bacterial diseases classified elsewhere |
| G02 | Meningitis in other infectious and parasitic diseases classified elsewhere |
| G03 | Meningitis due to other and unspecified causes |
| G04 | Encephalitis, myelitis and encephalomyelitis |
| G05* | Encephalitis, myelitis and encephalomyelitis in diseases classified elsewhere |
| G06 | Intracranial and intraspinal abscess and granuloma |
| G07* | Intracranial and intraspinal abscess and granuloma in diseases classified elsewhere |
| G08 | Intracranial and intraspinal phlebitis and thrombophlebitis |
| A17+ | Tuberculosis of nervous system |
| A81 | Atypical virus infections of central nervous system |
| A83 | Mosquito-borne viral encephalitis |
| A84 | Tick-borne viral encephalitis |
| A85 | Other viral encephalitis, not elsewhere classified |
| A86 | Unspecified viral encephalitis |
| A87 | Viral meningitis |
| A88 | Other viral infections of central nervous system, not elsewhere classified |
| A89 | Unspecified viral infection of central nervous system |

| Cardiovascular system infection |  |
| --- | --- |
| ICD-10-GM Codes: |  |
| I32 | Pericarditis in diseases classified elsewhere |
| I33 | Acute and subacute endocarditis |
| I39 | Endocarditis and heart valve disorders in diseases classified elsewhere |
| I40 | Acute myocarditis |
| I41 | Myocarditis in diseases classified elsewhere |
| I80 | Thombosis, phlebitis and thrombophlebitis |
| I38 | Endocarditis, valve unspecified |
| I98.1 | Cardiovascular disorders in other infectious and parasitic diseases classified elsewhere |

| Device-related infections |  |
| --- | --- |
| ICD-10-GM Codes: |  |
| T82.6 | Infection and inflammatory reaction due to cardiac valve prosthesis |
| T82.7 | Infection and inflammatory reaction due to other cardiac and vascular devices, implants and grafts |
| T83.5 | Infection and inflammatory reaction due to prosthetic device, implant and graft in urinary system |
| T83.6 | Infection and inflammatory reaction due to prosthetic device, implant and graft in genital tract |
| T84.5 | Infection and inflammatory reaction due to internal joint prosthesis |
| T84.6 | Infection and inflammatory reaction due to internal fixation device [any site] |
| T84.7 | Infection and inflammatory reaction due to other internal orthopaedic prosthetic devices, implants and grafts |
| T85.7 | Infection and inflammatory reaction due to other internal prosthetic devices, implants and grafts |

| Pregnancy associated infection |  |
| --- | --- |
| ICD-10-GM Codes: |  |
| O75.3 | Other infection during labour |
| O85 | Puerperal fever |
| O03.0 | Spontaneous abortion, complicated by genital tract and pelvic infection, incomplete |
| O03.5 | Spontaneous abortion, complicated by genital tract and pelvic infection, complete or unspecified |
| O04.0 | Medical abortion, complicated by genital tract and pelvic infection, incomplete |
| O04.5 | Medical abortion, complicated by genital tract and pelvic infection, complete or unspecified |
| O05.0 | Other abortion, complicated by genital tract and pelvic infectio, incomplete |
| O05.5 | Other abortion, complicated by genital tract and pelvic infection, complete or unspecified |
| O06.0 | Unspecified abortion, complicated by genital tract and pelvic infection, incomplete |
| O06.5 | Unspecified abortion, complicated by genital tract and pelvic infection, complete or unspecified |
| O07.0 | Failed medical abortion, complicated by genital tract and pelvic infection |
| O07.5 | Other and unspecified failed attempted abortion, complicated by genital tract and pelvic infection |
| O08.0 | Genital tract and pelvic infection following abortion and ectopic and molar pregnancy |
| O86 | Other puerperal infections |
| O23 | Infections of genitourinary tract in pregnancy |
| O41.1 | Infection of amniotic sac and membranes |
| O88.3 | Obstetric pyaemic and septic embolism |
| O91 | Infections of breast associated with childbirth |
| O98 | Maternal infectious and parasitic diseases classifiable elsewhere but complicating pregnancy, childbirth and the puerperium |

| Hospital-acquired infections |  |
| --- | --- |
| ICD-10-GM Codes: |  |
| T82.6 | Infection and inflammatory reaction due to cardiac valve prosthesis |
| T82.7 | Infection and inflammatory reaction due to other cardiac and vascular devices, implants and grafts |
| T84.5 | Infection and inflammatory reaction due to internal joint prosthesis |
| T84.6 | Infection and inflammatory reaction due to internal fixation device [any site] |
| T84.7 | Infection and inflammatory reaction due to other internal orthopaedic prosthetic devices, implants and grafts |
| T85.72 | Infection and inflammatory reaction due to internal prosthetic devices, implants and grafts in the central nervous system |
| T85.73 | Infection and inflammatory reaction due to prosthetic devices or implants of the mamma |
| T85.75 | Infection and inflammatory reaction due to internal prosthetic devices, implants or grafts of the hepatobiliary system or pancreas |
| T85.76 | Infection and inflammatory reaction due to internal prosthetic devices, implants or grafts of the other gastrointestinal system |
| T85.78 | Infection and inflammatory reaction due to other internal prosthetic devices, implants and grafts |
| O86.0 | Infection of obstetric surgical wound |
| T83.5 | Infection and inflammatory reaction due to prosthetic device, implant and graft in urinary system |
| T83.6 | Infection and inflammatory reaction due to prosthetic device, implant and graft in genital tract |
| A04.7 | Enterocolitis due to Clostridium difficile |
| U69.40! | Recurrent infection due to Clostridium difficile |
| T80.2 | Infections following infusion transfusion and therapeutic injection |
| T82.7 | Infection and inflammatory reaction due to other cardiac and vascular devices, implants and grafts |
| T81.4 | Infection following a procedure, not elsewhere classified |
| T85.71 | Infection and inflammatory reaction due to peritoneal dialysis catheter |
| T85.74 | Infection and inflammatory reaction due to percutaneous endoscopic gastrostomy/jejunostomy, T88.0 |
| U69.00 | Hospital-acquired pneumonia in patients aged 18 years or older |

For the identification of catheter-related infections, ICD-10 codes T80.2 and T82.7 had to be coded together with the operation and procedure codes 8-831, 5-399.5, 5-399.6, 5-399.7 for the implantation or revision of central-venous catheter systems.

| Multidrug-resistant infections |  |
| --- | --- |
| ICD-10-GM Codes: |  |
| U80.! | Grampositive bacteria with specified antibiotic resistance, requiring special therapeutic or hygienic measures |
| U81.! | Gram negative bacteria with specified antibiotic resistanc, requiring special therapeutic or hygienic measures |
| U82.! | Mycobacteria with resistance against TB drugs (first line) |
| U83.! | Candida with resistance against Fluconazole and Voriconazole |
| U84.! | Herpes virus with restistance against antivirals |
| U85! | Human Immunodeficiency Virus with resistance against antivirals or proteinase â€“ inhibitors |
| OPS Codes: |  |
| 8-987 | Complex treatment in the case of colonisation or infection with multidrug-resistant pathogens [MDR] |
|  |  |

| Severe sepsis |  |
| --- | --- |
| ICD-10-GM Codes: |  |
| R65.1 | Systemic Inflammatory Response Syndrome of infectious origin with organ failure |
| R57.2 | Septic shock |

| Septic shock |  |
| --- | --- |
| ICD-10-GM Codes: |  |
| R57.2 | Septic shock |

| ICU Treatment |  |
| --- | --- |
| OPS Codes: |  |
| 8-980 | Intensive care complex treatment |
| 8-98f | Costly intensive care complex treatment (basic procedure) |
| 8-98d | Intensive care complex treatment in childhood (basic procedure) |
| 8-98c | Intensive care complex treatment in childhood |

| Mechanical Ventilation |  |
| --- | --- |
| OPS Codes: |  |
| 8-713 | Mechanical ventilation and respiratory support in adults |
| 8-712 | Mechanical ventilation and respiratory support in children and adolescents |
| 8-714 | Special procedure for mechanical ventilation in the case of severe respiratory failure |
| 8-70 | Access for mechanical ventilation and measures to maintain the airway |
| 8-71 | Mechanical ventilation and respiratory support via a mask or tube and ventilation weaning |

| Renal replacement therapy |  |
| --- | --- |
| OPS Codes: |  |
| 8-853 | Haemofiltration |
| 8-854 | Haemodialysis |
| 8-855 | Haemodiafiltration |
| 8-857 | Peritoneal dialysis |
| 8-85a | Dialysis procedure due to a functional failure and failure of a kidney transplant |

| Surgical treatment |  |
| --- | --- |
| OPS Codes: |  |
| Any OPS Code from Chapter 5 |  |

| Amputation during index hospitalization |  |
| --- | --- |
| OPS Codes: |  |
| 5-862 | Amputation and exarticulation of upper extremity |
| 5-863 | Amputation and exarticulation of hand |
| 5-864 | Amputation and exarticulation of lower extremity |
| 5-865 | Amputation and exarticulation of foot |
| 5-866 | Revision of amputation area |

| **Discharge disposition** | **Definition** |
| --- | --- |
| regular | regular termination of treatment, with or without post-discharge treatment intended |
| other hospital | transfer to another hospital;  transfer to another hospital as part of a cooperation;  external transfer for psychiatric treatment |
| hospice | discharge into a hospice |
| rehabilitation | discharge into a rehabilitation facility |
| nursing home | discharge into a long-term care facility |
| other | treatment terminated for other reasons, with or without post-discharge treatment intended;  Treatment terminated against medical advice, with or without post-discharge treatment intended;  Change of responsibility of the cost bearer;  Death;  internal routing;  Treatment terminated for other reasons, post-inpatient treatment  intended;  external transfer with relocation or change between the  Remuneration ranges of the DRG flat rate case, according to section 17b (1) first sentence of the Hospital Funding Act;  Internal transfer with a change between the DRG fee ranges  according to section 17b (1)first sentence of the Hospital Funding Act;  Relocation;  Discharge before resumption with reclassification;  Discharge before resumption with reclassification due to complication;  Discharge or transfer with subsequent readmission;  Case closure (internal transfer) when changing between full,  day-care and ward-equivalent treatment;  Start of an outside stay with an absence past midnight  (BPflV area - for the specialist department for laying);  Ending an outside stay with an absence past midnight  (BPflV area - for pseudo specialist department 0003);  Discharge at the end of the year if accepted in the previous year (for the purposes of  Billing - § 4 PEPPV);  Beginning of a period without direct patient contact  (station equivalent treatment);  Termination of a period without direct patient contact  (ward equivalent treatment - for pseudo-specialist department 0004); |

| Level of care in the year prior to index hospitalization | A care level/grade describes a person's individually recognized need for care and assistance in accordance with the German Care Insurance Act (SGB XI) and thus determines the scope of support benefits that could be applied for from the relevant care insurance fund. |
| --- | --- |

| Nursing home residence in pre-observation period |  |
| --- | --- |
|  | Nursing home placement: yes |

New cognitive, psychological and medical impairments were defined according to the SEPFROK study definition^5^

| **Cognitive impairment** |  |
| --- | --- |
| ICD-10-GM Codes: |  |
| F06.7 | Mild cognitive disorder |
| U51.- | Impairment of cognitive function |
| R41.0 | Disorientation, unspecified |
| F00* | Dementia in Alzheimer disease |
| F01 | Vascular dementia |
| F02* | Dementia in other diseases classified elsewhere |
| F03 | Unspecified dementia |
| F04 | Organic amnesic syndrome, not induced by alcohol and other psychoactive substances |
| F05 | Delirium, not induced by alcohol and other psychoactive substances |
| F06.9 | Unspecified organic mental disorder due to brain damage and dysfunction and to physical disease |
| F07.8 | Other organic personality and behavioural disorders due to brain disease, damage and dysfunction |
| F07.9 | Unspecified organic personality and behavioural disorder due to brain disease, damage and dysfunction |
| G30 | Alzheimer disease |
| G31.0 | Circumscribed brain atrophy |
| G31.1 | Senile degeneration of brain, not elsewhere classified |
| G31.9 | Degenerative disease of nervous system, unspecified |
| G32* | Other degenerative disorders of nervous system in diseases classified elsewhere |

| **Psychological impairment** |  |
| --- | --- |
| PTSD |  |
| ICD-10-GM Codes: |  |
| F43 | Reaction to severe stress, and adjustment disorders |
| F43.0 | Acute stress reaction |
| F43.1 | Post-traumatic stress disorder |
| F43.2 | Adjustment disorders |
| F43.8 | Other reactions to severe stress |
| F43.9 | Reaction to severe stress, unspecified |
| Depression |  |
| ICD-10-GM Codes: |  |
| F32 | Depressive episode |
| F33 | Recurrent depressive disorder |
| F34.1 | Dysthymia |
| F38 | Other mood [affective] disorders |
| F41.2 | Mixed anxiety and depressive disorder |
| F06.3 | Organic mood [affective] disorders |
| Anxiety |  |
| ICD-10-GM Codes: |  |
| F40 | Phobic anxiety disorders |
| F41 | Other anxiety disorders |
| F06.4 | Organic anxiety disorder |
| Sleeping disorders |  |
| ICD-10-GM Codes: |  |
| F51 | Nonorganic sleep disorders |
| G47 | Sleep disorders |
| Substance abuse |  |
| ICD-10-GM Codes: |  |
| F10 | Mental and behavioural disorders due to use of alcohol |
| F11 | Mental and behavioural disorders due to use of opioids |
| F12 | Mental and behavioural disorders due to use of cannabinoids |
| F13 | Mental and behavioural disorders due to use of sedatives or hypnotics |
| F14 | Mental and behavioural disorders due to use of cocaine |
| F15 | Mental and behavioural disorders due to use of other stimulants, including caffeine |
| F16 | Mental and behavioural disorders due to use of hallucinogens |
| F17 | Mental and behavioural disorders due to use of tobacco |
| F18 | Mental and behavioural disorders due to use of volatile solvents |
| F19 | Mental and behavioural disorders due to multiple drug use and use of other psychoactive substances |

| **Medical impairment** |  |
| --- | --- |
| Respiratory dysfunction |  |
| ICD-10-GM Codes: |  |
| J96 | Respiratory failure, not elsewhere classified |
| J98 | Other respiratory disorders |
| R06.0 | Dyspnoea |
| J80 | Adult respiratory distress syndrome |

| Cardiovascular diseases |  |
| --- | --- |
| Coronary heart disease and myocardial infarction |  |
| ICD-10-GM Codes: |  |
| I20 | Angina pectoris |
| I21 | Acute myocardial infarction |
| I22 | Subsequent myocardial infarction |
| I24 | Other acute ischaemic heart diseases |
| I25 | Chronic ischaemic heart disease |
| Cardiomyopathy |  |
| ICD-10-GM Codes: |  |
| I42 | Cardiomyopathy |
| Heart failure |  |
| ICD-10-GM Codes: |  |
| I50 | Heart failure |
| Cardiac arrhythmias |  |
| ICD-10-GM Codes: |  |
| I47 | Paroxysmal tachycardia |
| I48 | Atrial fibrillation and flutter |
| I49 | Other cardiac arrhythmias |

| Cerebrovascular diseases |  |
| --- | --- |
| ICD-10-GM Codes: |  |
| I63 | Cerebral infarction |
| I64 | Stroke, not specified as haemorrhage or infarction |
| I65 | Occlusion and stenosis of precerebral arteries, not resulting in cerebral infarction |
| I66 | Occlusion and stenosis of cerebral arteries, not resulting in cerebral infarction |

| Renal diseases |  |
| --- | --- |
| ICD-10-GM Codes: |  |
| N17 | Acute renal failure |
| N18 | Chronic kidney disease |
| N19 | Unspecified kidney failure |

| Hepatic diseases |  |
| --- | --- |
| ICD-10-GM Codes: |  |
| K72.1 | Chronic hepatic failure |

| Metabolic diseases |  |
| --- | --- |
| Diabetes mellitus |  |
| ICD-10-GM Codes: |  |
| E11 | Type 2 diabetes mellitus |
| E12 | Malnutrition-related diabetes mellitus |
| E13 | Other specified diabetes mellitus |
| E14 | Unspecified diabetes mellitus |
| Other metabolic diseases |  |
| ICD-10-GM Codes: |  |
| E27 | Other disorders of adrenal gland |
| E35* | Disorders of endocrine glands in diseases classified elsewhere |
| E34.9 | Endocrine disorder, unspecified |
| E23 | Hypofunction and other disorders of pituitary gland |

| Anaemia |  |
| --- | --- |
| ICD-10-GM Codes: |  |
| D50 | Iron deficiency anaemia |
| D51 | Vitamin B12 deficiency anaemia |
| D52 | Folate deficiency anaemia |
| D53 | Other nutritional anaemias |
| D63 | Anaemia in chronic diseases classified elsewhere |
| D64.9 | Anaemia, unspecified |

| Neuromuscular/musculoskeletal diseases |  |
| --- | --- |
| ICUAW/CIP/CIM |  |
| ICD-10-GM Codes: |  |
| G62.8 | Critical illness polyneuropathy |
| G72.8 | Critical illness myopathy |
| Dysphagia |  |
| ICD-10-GM Codes: |  |
| R13 | Dysphagia |
| Voice disorders |  |
| ICD-10-GM Codes: |  |
| R49 | Voice disturbances |
| Contractures |  |
| ICD-10-GM Codes: |  |
| M62.4 | Contracture of muscle |
| M24.5 | Contracture of joint |
| M25.6 | Stiffness of joint, not elsewhere classified |
| M21.62 | Acquired Pes equinus |
| Immobility |  |
| ICD-10-GM Codes: |  |
| R26.2 | Difficulty in walking, not elsewhere classified |
| R26.3 | Immobility |
| R29.6 | Tendency to fall, not elsewhere classified |
| Z99.3 | Dependence on wheelchair |
| Z74.0 | Need for assistance due to reduced mobility |

| Decubitus |  |
| --- | --- |
| ICD-10-GM Codes: |  |
| L89 | Decubitus ulcer and pressure area |

| Complications of tracheostomy |  |
| --- | --- |
| ICD-10-GM Codes: |  |
| Z43.0 | Attention to tracheostomy |
| Z93.0 | Tracheostomy status |
| J95.0 | Tracheostomy malfunction |

| Tracheal stenoses |  |
| --- | --- |
| ICD-10-GM Codes: |  |
| J95.5 | Postprocedural subglottic stenosis |
| J95.81 | Tracheal stenosis following a procedure |
| J38.6 | Stenosis of larynx |
| J39.8 | Acquired tracheal stenosis |

| Urogenital diseases |  |
| --- | --- |
| Incontinence |  |
| ICD-10-GM Codes: |  |
| R32 | Unspecified urinary incontinence |
| N39.3 | Stress incontinence |
| N39.4 | Other specified urinary incontinence |
| R15 | Faecal incontinence |
| Sexual disorders |  |
| ICD-10-GM Codes: |  |
| F52 | Sexual dysfunction, not caused by organic disorder or disease |
| Urethral stricture |  |
| ICD-10-GM Codes: |  |
| N99.1 | Postprocedural urethral stricture |

| Sensory disorders |  |
| --- | --- |
| Vestibular disorders |  |
| ICD-10-GM Codes: |  |
| R42 | Dizziness and giddiness |
| Hearing disorder |  |
| ICD-10-GM Codes: |  |
| H90 | Conductive and sensorineural hearing loss |
| H91 | Other hearing loss |
| H93 | Other disorders of ear, not elsewhere classified |
| Taste and smelling disorders |  |
| ICD-10-GM Codes: |  |
| R43 | Disturbances of smell and taste |

| Impairment of nutrition |  |
| --- | --- |
| ICD-10-GM Codes: |  |
| E41 | Nutritional marasmus |
| E43 | Unspecified severe protein-energy malnutrition |
| E44 | Protein-energy malnutrition of moderate and mild degree |
| E46 | Unspecified protein-energy malnutrition |
| R63.0 | Anorexia |
| R63.3 | Feeding difficulties and mismanagement |
| R63.4 | Abnormal weight loss |
| R63.6 | Insufficient intake of food and water |
| R63.8 | Other symptoms and signs concerning food and fluid intake |
| R64 | Cachexia |

| Multidrug-resistant infections |  |
| --- | --- |
| ICD-10-GM Codes: |  |
| U80.-! | Gram-positive bacteria with specified antibiotic resistance requiring special therapeutic or hygienic measures |
| U81.-! | Gram-negative bacteria with specified antibiotic resistance requiring special therapeutic or hygienic measures |
| U82 | Mycobacteria with resistance against TB drugs (first line) |
| U83 | Candida with resistance against Fluconazole and Voriconazole |
| U84 | Herpes virus with restistance against antivirals |

| Chronic pain |  |
| --- | --- |
| ICD-10-GM Codes: |  |
| R52.1 | Chronic intractable pain |
| R52.2 | Other chronic pain |
| R52.9 | Pain unspecified |
| F45.4 | Persistent somatoform pain disorder |
| F45.41 | Chronic pain disorder associated with psychological and behavioural factors |
| G54.6 | Phantom limb syndrome with pain |

| Fatigue |  |
| --- | --- |
| ICD-10-GM Codes: |  |
| R53 | Malaise and fatigue |
| G93.3 | Chronic fatigue syndrome |

**Table 1: Patient demographics and clinical features of the sepsis survivor cohort**

| **N 12-months survivors** | 7.370 |
| --- | --- |
| Age, mean (SD); median (IQR) | 48.8 (9.8); 52 (12) |
| Female sex, % (95% CI) | 35.3 (34.2 - 36.4) |
|  |  |
| **Comorbidities in the 12 months prior to hospital admission** |  |
| CCI, mean (SD); median (IQR) | 1.4 (1.5); 1 (2) |
| CCI = 0, % (95% CI) | 35.7 (34.4 - 37.0) |
| CCI = 1, % (95% CI) | 26.5 (25.3 - 27.8) |
| CCI = 2-4, % (95% CI) | 33.5 (32.2 - 34.7) |
| CCI >4, % (95% CI) | 4.3 (3.0 - 5.6) |
|  |  |
| Diabetes, % (95% CI) | 18.5 (17.7 - 19.4) |
| Chronic pulmonary disease, % (95% CI) | 24.3 (23.4 - 25.3) |
| Renal disease, % (95% CI) | 9.6 (9.0 - 10.3) |
| Congestive heart failure and myocardial infarction, % (95% CI) | 10.8 (10.1 - 11.5) |
| Cancer, % (95% CI) | 16.5 (15.6 - 17.3) |
| Dementia, % (95% CI) | 1.5 (1.3 - 1.9) |
| Cerebrovascular disease, % (95% CI) | 6.5 (5.9 - 7.0) |
| Liver disease, % (95% CI) | 14.8 (14.0 - 15.6) |
| HIV/AIDS, % (95% CI) | 0.5 (0.4 - 0.7) |
|  |  |
| **Index hospitalization** |  |
| Admission as emergency, % (95% CI) | 55.4 (54.3 - 56.6) |
| Hospital length of stay, mean (SD); median (IQR) | 22 (24.4); 14 (19) |
|  |  |
| Focus of infection, % (95% CI) |  |
| - Respiratory tract | 32.6 (31.5 - 33.7) |
| - Abdominal | 15.5 (14.6 - 16.3) |
| - Wound/soft tissue infection | 9.6 (9.0 - 10.3) |
| - Genitourinary system | 24.3 (23.4 - 25.3) |
| - Central nervous system | 1.9 (1.6 - 2.2) |
| - Cardiovascular system | 4.1 (3.7 - 4.6) |
| - Device-related | 8.0 (7.4 - 8.6) |
| - Pregnancy associated infection | 0.7 (0.5 - 0.9) |
| Hospital-acquired infection, % (95% CI) | 19.1 (18.3 - 20.1) |
| Multi-resistant infection, % (95% CI) | 3.3 (2.9 - 3.8) |
| Occurance of severe sepsis, % (95% CI) | 30.6 (29.6 - 31.7) |
| Occurance of septic shock, % (95% CI) | 8.0 (7.4 - 8.6) |
| ICU treatment, % (95% CI) | 32.7 (31.7 - 33.8) |
| Mechanical ventilation, % (95% CI) | 19.8 (18.9 - 20.7) |
| Renal replacement therapy, % (95% CI) | 5.8 (5.3 - 6.4) |
| Surgical treatment, % (95% CI) | 39.1 (38.0 - 40.3) |
| Amputation during treatment, % (95% CI) | 1.3 (1.1 - 1.6) |
|  |  |
|  |  |
| Discharge disposition of survivors, % |  |
| - regular discharge | 80.1 (79.2 - 81) |
| - other hospital | 11.1 (10.2 - 12) |
| - rehabilitation | 5.1 (4.2 - 6) |
| - nursing home | 1.6 (0.8 - 2.5) |
| - hospice | 0 (0 - 0.9) |
| - other | 2 (1.2 - 2.9) |

Abbreviations: CCI = Charlson Comorbidity Index (unweighted), CI = Confidence Interval, ICU = Intensive Care Unit, IQR = Interquartile Range, SD = Standard Deviation
